# *Rickettsia* and Vector Biodiversity of Spotted Fever Focus, Atlantic Rain Forest Biome, Brazil

**DOI:** 10.3201/eid2003.131013

**Published:** 2014-03

**Authors:** Nicole O. Moura-Martiniano, Erik Machado-Ferreira, Karen M. Cardoso, Flávia S. Gehrke, Marinete Amorim, Andréa C. Fogaça, Carlos A.G. Soares, Gilberto S. Gazêta, Teresinha T.S. Schumaker

**Affiliations:** Universidade de São Paulo, São Paulo, Brazil (N.O. Moura-Martiniano, K.M. Cardoso, F.S. Gehrke, A.C. Fogaça, T.T.S. Schumaker);; Universidade Federal do Rio de Janeiro, Rio de Janeiro, Brazil (E. Machado-Ferreira, C.A.G. Soares);; Fundação Oswaldo Cruz, Rio de Janeiro (M. Amorim, G.S. Gazêta)

**Keywords:** Tick. flea. Rickettsia. spotted fever, vector-borne infections, Brazil

**To the Editor:** *Rickettsia rickettsii*, *R. felis*, and *R. parkeri*, strain Atlantic rainforest, have been characterized after being found in areas to which Brazilian spotted fever (BSF) is endemic (*1*,*2*), which indicates the complexity of their epidemic and enzootic cycles. The Atlantic rain forest is one of the largest and richest biomes of Brazil, and antropic action has intensely influenced its transformation. Most BSF cases and all BSF-related deaths are recorded in this biome area.

Many BSF cases were recorded in Paraíba do Sul river basin, one of the most urbanized and industrialized areas of Brazil. To better understand arthropod and *Rickettsia* diversity in this area,, we analyzed 2,076 arthropods from Rio de Janeiro state, Atlantic rain forest biome.

During October 2008–November 2009, we collected ticks and fleas from hosts and environments in 7 cities where high numbers of BSF cases were recorded (Rio de Janeiro State Health Secretary, unpub. data) and where fisiogeographic characteristics differed. After morphologic classification (*3*), the arthropods were individually separated or grouped by sex, developmental stage, and host for total DNA extraction (*4*).

We used 2 *Rickettsia*-specific primer sets (CS2–78/CS2–323 and CS4–239/CS4–1069) to amplify 401 bp and 834 bp, respectively, of the citrate synthase gene (*gltA*) (*5*,*6*). Presumptive *Rickettsia*-positive samples were tested for spotted fever group (SFG)–specific primer set *Rr*190.70p/*Rr*190.602n for 532 bp from the *ompA* gene (*7*). *R. rickettsii* DNA and bi-distilled water were used as positive and negative controls, respectively. PCR products were purified (NucleoSpin Extract II kit; Macherey-Nagel, Düren, Germany), cloned (pTZ57R/T; Fermentas-Thermo Fisher Scientific, Waltham, MA, USA), and sequenced by using specific vector primer sets (BigDye Reaction kit, Applied Biosystems, Foster City, CA, USA). Sequences were edited by using SeqMan program (Lasergene 10.1; DNASTAR Inc., Madison, WI, USA), and similarities were obtained by BLAST analysis (http://blast.ncbi.nlm.nih.gov). The phylogenies were assessed by applying neighbor-joining and maximum-parsimony methods, with the Kimura 2-parameter correction model. We used ClustalW 2.1 (www.clustal.org) to align sequences and produced phylogenetic trees by using 1,000 replicates bootstrap in MEGA 5.0 software (www.megasoftware.net).

We collected and analyzed ticks of the following species: *Amblyomma cajennense* (1,723 ticks), *Rhipicephalus sanguineus* (109), *Anocentor nitens* (63), *Boophilus microplus* (33), *Amblyomma aureolatum* (2), and *Amblyomma dubitatum* (2). We collected and analyzed *Ctenocephalides felis* (143 fleas) and *C. canis* (1) fleas.

PCR analysis showed *Rickettsia* DNA in 11 individual or pooled samples. This finding indicated minimal infection rates of 0.2% (4/1,723) for *A. cajennense* ticks, 50% (2/4) for *A. dubitatum* ticks, 3.0% (1/33) for *B. microplus* ticks, 100% (1/1) for *C. canis* fleas, and 2.8% (4/143) for *C. felis* fleas. Expected amplicon size, determined by using the *gltA* 401-bp primer set, was observed for all positive samples. Two were also positive by PCR for *gltA* 834 bp and 4 for *ompA* primer set (Technical Appendix). The sequences were deposited in GenBank; BLASTn analysis (http://blast.ncbi.nlm.nih.gov/blast.cgi) indicates that these sequences belong to AG (ancestral) or SFG rickettsiae (Figure).

**Figure:**
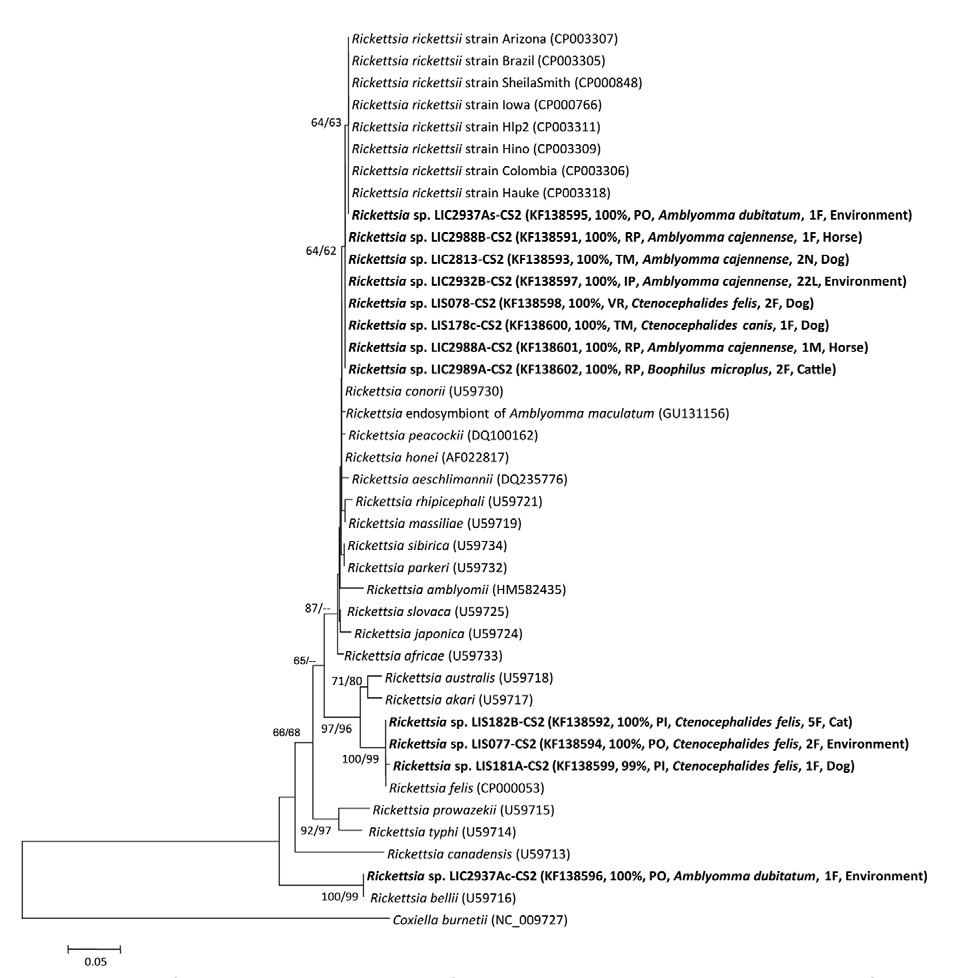
Phylogenetic inferences by neighbor-joining method from 1,000 replicated trees based on partial sequence of the *Rickettsia* *gltA* gene (CS2 401 bp). Evolutionary distances were estimated by the Kimura 2-parameter model. Bootstrap values >60% are shown (neighbor-joining/maximum-parsimony). Sequences obtained are in boldface, and GenBank accession numbers are in parentheses, followed by the similarity percentage (BLAST, http://blast.ncbi.nlm.nih.gov), the locality acronym (PO, Porciúncula; RP, São José do Vale do Rio Preto; TM, Trajano de Moraes; IP, Itaperuna; VR, Volta Redonda; PI, Piraí), the arthropod vector species, the composition of the sample (L, larvae; N, nymph; F, female; M, male), and the host. Scale bar indicates nucleotide substitutions per site.

In phylogenetic inferences, 8 samples were grouped with SFG *R. rickettsii*, supported by bootstrap value >62%. In addition, 3 samples were closely related to SFG *R. felis,* strongly supported by bootstrap values >99%; *Rickettsia* sp. LIC2937Ac was closely related to AG *R. bellii* under a bootstrap support >99% (Figure).

Epidemic manifestations of rickettsial diseases vary by ecotope characteristics, human activity, and vector bioecology in natural foci. BSF is a clinically distinct rickettsial infection in foci to which it is endemic. BSF-related illness and death vary by the *Rickettsia* species that can coexist in a given area and that can share or not share epidemiologic elements.

Molecular identification of *R. rickettsii* in *A. cajennense* ticks was recorded only in the Paraíba do Sul River basin of southeastern Brazil (*8*), as confirmed in our study. This eco-epidemiologic aspect, its great anthropophily, and its presence in all municipalities surveyed, with absolute frequency greater than other species, demonstrates the possible effect of this tick on epidemic cycle development for the analyzed region, which does not seem to occur in other regions.

*R. rickettsii* infection of *A. dubitatum* ticks in the 1 focus analyzed might indicate its relevance in specific epidemiologic scenarios. We detected highly similar sequences of different species of *Rickettsia* (LIC2937A) in the same *A. dubitatum* tick specimen (Figure). Other studies have recorded multiple *Rickettsia* infections in 1 tick specimen (*9*,*10*).

Our finding of *C. felis* fleas in 6 of the 7 outbreaks investigated highlights the possible role of this flea in maintaining *Rickettsia* in Rio de Janeiro state. *C. felis* and *C. canis* fleas infected with *R. rickettsii* seem to confirm this potential. Nevertheless, the real epidemiologic value of this report in the BSF cycle deserves to be further investigated.

Our results indicate that dogs and horses are the primary vertebrates in the *Rickettsia* enzootic cycle in the investigated focus, and, considering their common presence in human environments, they must be important in maintaining possible rickettsial vectors to humans. These results contribute to the mapping of BSF-endemic areas and to the understanding of the circulation and epidemiology of *Rickettsia* sp. in an area with one of the highest fatal concentrations of BSF.

Technical Appendix Phylogenetic inferences by neighbor-joining method from 1,000 replicated trees based on partial sequence of the *gltA* and *ompA* genes.
