# Supplementary material for: Rickettsia and Vector Biodiversity of Spotted Fever Focus, Atlantic Rain Forest Biome, Brazil
Source: Emerg Infect Dis. 2014 Mar;20(3):498–500. doi: 10.3201/eid2003.131013 (PMC3944845; doi:10.3201/eid2003.131013)
Supplement: Technical Appendix — Phylogenetic inferences by neighbor-joining method from 1,000 replicated trees based on partial sequence of the gltA and ompA genes. [file 13-1013-Techapp-s1.pdf]

Article DOI: http://dx.doi.org/10.3201/eid2003.131013

Rickettsial and Vector Biodiversity of Spotted Fever Focus, Atlantic Rain Forest Biome, Brazil

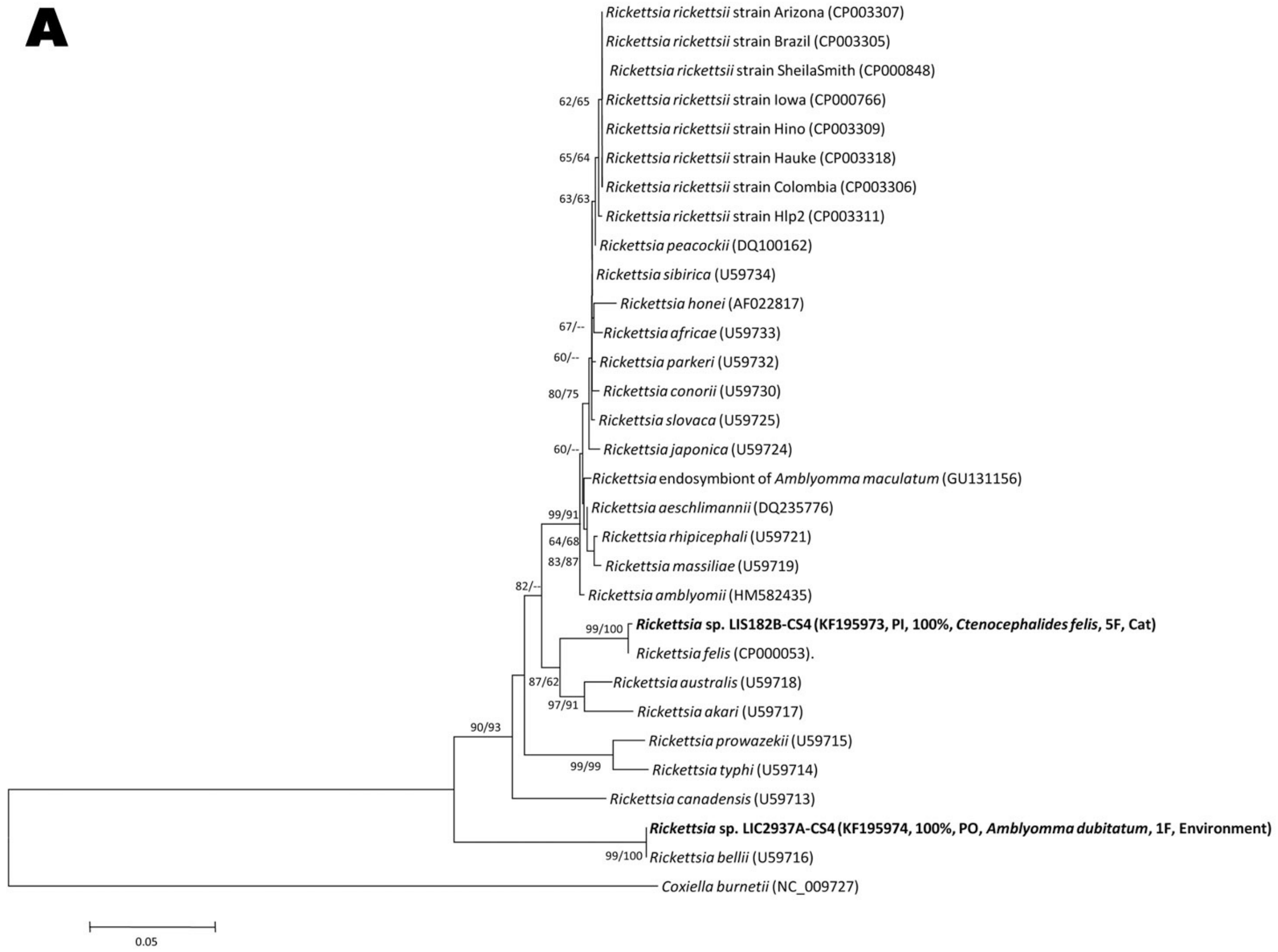

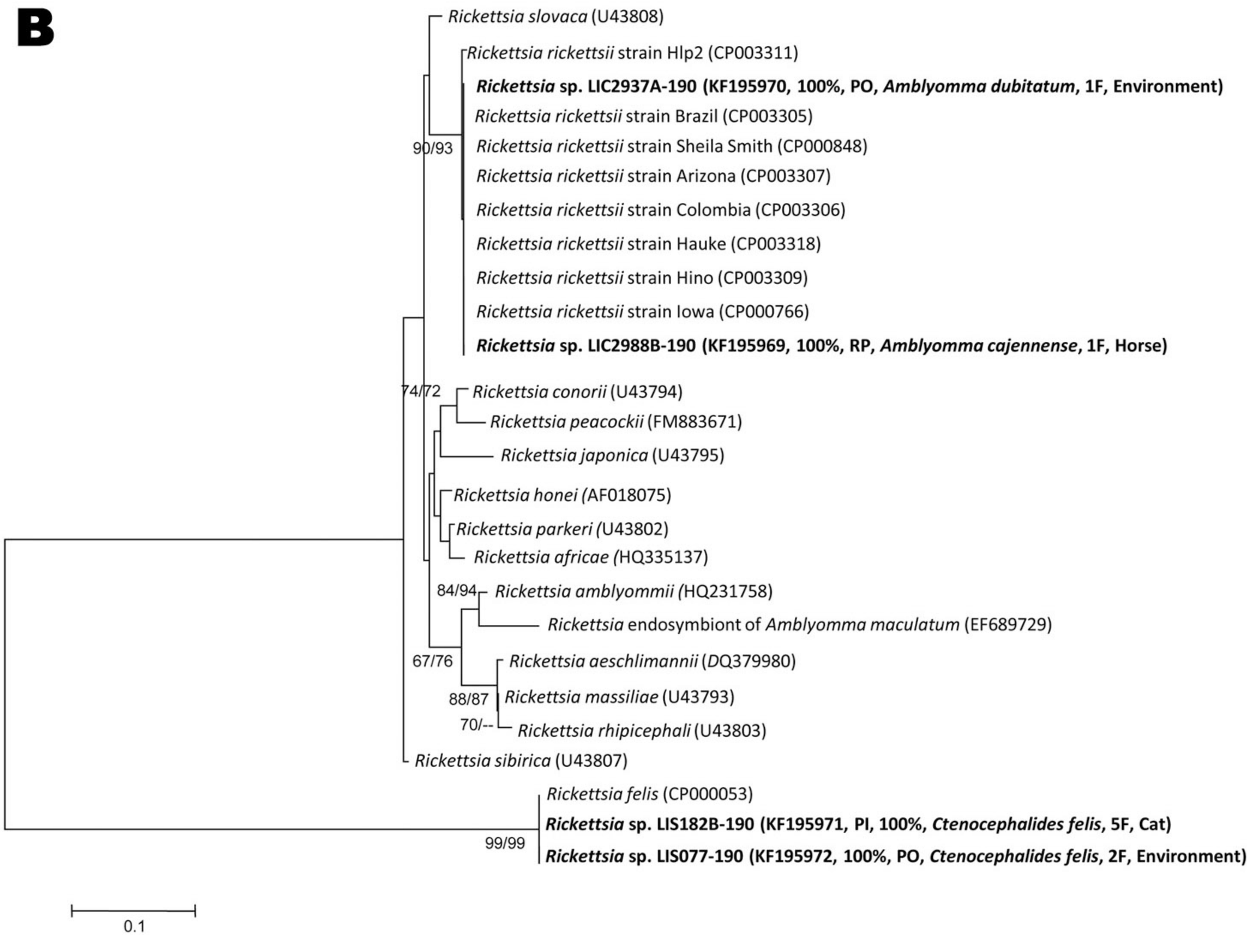

Technical Appendix

Technical Appendix Figure. A) Phylogenetic inferences by neighbor-joining method from 1,000 replicated trees based on partial sequence of the *gltA* gene (CS4 834 bp). Evolutionary distances were estimated by Kimura 2-parameter model. Bootstrap values >60% are shown (neighbor-joining/maximum parsimony). Sequences obtained are in bold, and the GenBank accession numbers are in parentheses, followed by the similarity percentage (BLAST, http://blast.ncbi.nlm.nih.gov), the locality acronym (PO, Porciúncula; PI, Piraí), the arthropod vector species, the composition of the sample (F, female) and the host. B) Phylogenetic inferences by neighbor-joining method from 1,000 replicated trees based on partial sequence of the *ompA* gene. Evolutionary distances were estimated by using the Kimura 2-parameter model. Bootstrap values >60% are shown (neighbor-joining/maximum-parsimony). Sequences obtained are presented in bold, and the GenBank accession numbers are in parentheses, followed by the similarity percentage (BLAST), the locality acronym (PO, Porciúncula; RP, São José do Vale do Rio Preto; PI, Piraí), the arthropod vector species, the composition of the sample (F, female), and the host. Scale bar indicates nucleotide substitutions per site.
